# Temporary sequestration of cholesterol and phosphatidylcholine within extracellular domains of ABCA1 during nascent HDL generation

**DOI:** 10.1038/s41598-018-24428-6

**Published:** 2018-04-18

**Authors:** Masato Ishigami, Fumihiko Ogasawara, Kohjiro Nagao, Hidehiko Hashimoto, Yasuhisa Kimura, Noriyuki Kioka, Kazumitsu Ueda

**Affiliations:** 1 Division of Applied Life Sciences, Graduate School of Agriculture, Kyoto University, Kyoto, 606-8502 Japan; 2 Department of Synthetic Chemistry and Biological Chemistry, Graduate School of Engineering, Kyoto University, Kyoto, 615-8510 Japan; 3 Institute for Integrated Cell-Material Sciences (WPI-iCeMS), Kyoto University, Kyoto, 606-8502 Japan

## Abstract

The quality and quantity of high-density lipoprotein (HDL) in blood plasma are important for preventing coronary artery disease. ATP-binding cassette protein A1 (ABCA1) and apolipoprotein A-I (apoA-I) play essential roles in nascent HDL formation, but controversy persists regarding the mechanism by which nascent HDL is generated. In the “direct loading model”, apoA-I acquires lipids directly from ABCA1 while it is bound to the transporter. By contrast, in the “indirect model”, apoA-I acquires lipids from the specific membrane domains created by ABCA1. In this study, we found that trypsin treatment causes rapid release of phosphatidylcholine (PC) and cholesterol from BHK/ABCA1 cells, and that the time course of lipid release coincides with those of trypsin digestion of extracellular domains (ECDs) of surface ABCA1 and of release of ECD fragments into the medium. This trypsin-dependent lipid release was dependent on ABCA1 ATPase activity, and did not occur in cells that express ABCG1, which exports lipids like ABCA1 but does not have large ECDs. These results suggest that the trypsin-sensitive sites on the cell surface are the large ECDs of ABCA1, and that lipids transported by ABCA1 are temporarily sequestered within the ECDs during nascent HDL formation.

## Introduction

The quality and quantity of high-density lipoprotein (HDL) in blood plasma are important for preventing coronary artery disease^1^. ATP-binding cassette protein A1 (ABCA1) exports excess cellular cholesterol and phosphatidylcholine (PC) to lipid-free apolipoprotein A-I (apoA-I) in serum^2^, thereby generating nascent HDL, a bilayer fragment consisting of 200–700 lipids wrapped by two to four molecules of apoA-I^3,4^. This step of nascent discoidal HDL generation is critical for HDL formation, as demonstrated by the fact that missense mutations in ABCA1 cause Tangier disease, a condition in which patients have very low or no circulating HDL^5–8^.

ABCA1 contains a tandem repeat of structural halves consisting of six transmembrane (TM) helices followed by a nucleotide-binding domain (NBD). ABCA1 has two large characteristic extracellular domains (ECDs), one between TM1 and TM2 and the other between TM7 and TM8^9–12^. The ECDs consist of more than 900 amino-acid residues, and constitute almost half of the ABCA1 molecule. Two intramolecular disulfide bonds are formed between these domains, and they are necessary for apoA-I binding and HDL formation^13^. Several pieces of evidence indicate that apoA-I interacts directly with a specific conformation of the ECDs that forms in an ATP-dependent manner. Chemical cross-linkers can cross-link apoA-I with ABCA1^12,14,15^, and ATPase-deficient ABCA1 mutants fail to mediate apoA-I binding and crosslinking^16^. The ECDs undergo conformational changes in response to ATP hydrolysis by ABCA1, which is associated with apoA-I binding^17^. Furthermore, single-molecule imaging using total internal reflection fluorescence (TIRF) microscopy revealed that a direct interaction forms between apoA-I and ABCA1 on the plasma membrane during the initial step of HDL formation^18^.

Two models have been proposed to explain the mechanism of nascent HDL generation^19^. In the direct loading model, apoA-I acquires lipids directly from ABCA1 while it is bound to the transporter, whereas in the indirect model, it acquires lipids from the specific membrane domains created by the phospholipid translocation activity of ABCA1. The latter model is supported by the existence of two types of apoA-I binding sites on the plasma membranes of cells expressing ABCA1, namely a high-affinity/low-capacity binding site and a low-affinity/high-capacity binding site^20,21^. It is also supported by the observation that apoA-I, by itself, can bind to liposomes with high curvature and form discoidal HDL particles spontaneously *in vitro*^22^.

In this study, we found that trypsin treatment causes the rapid release of PC and cholesterol from baby hamster kidney (BHK)/ABCA1 cells, suggesting that PC and cholesterol are temporarily sequestered at trypsin-sensitive sites on the surface of cells in an ATP-dependent manner. These results suggest that the trypsin-sensitive sites on the cell surface are the large ECDs of ABCA1, and that lipids transported by ABCA1 are temporarily sequestered within the ECDs during nascent HDL formation.

## Results

### Trypsin treatment of BHK/ABCA1 cells causes PC and cholesterol release into the medium

**Figure 1:**
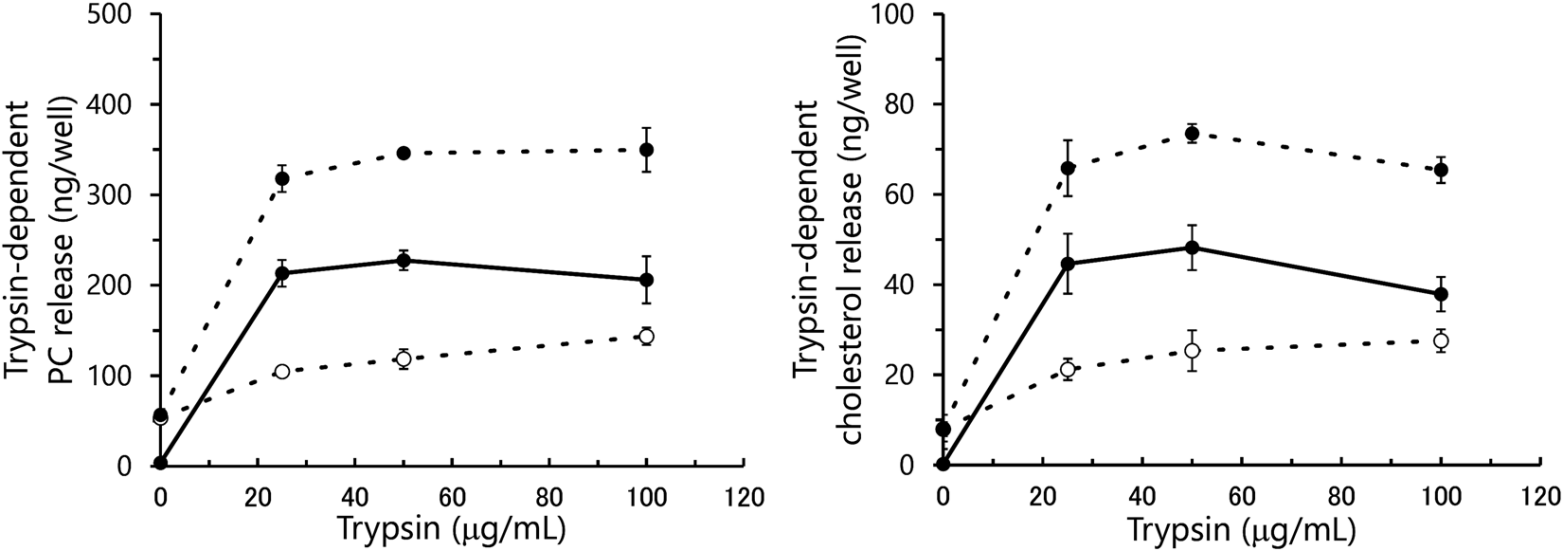
Trypsin-dependent PC and cholesterol release from BHK/ABCA1 cells. BHK/Mock (○, dotted line) and BHK/ABCA1 (●, dotted line) cells were incubated with 0, 25, 50, or 100 μg/mL of trypsin for 60 min at 37 °C. The amounts of PC and cholesterol released into the medium were measured. ABCA1-specific release of PC and cholesterol (●, solid line) were calculated by subtracting the release from BHK/Mock cells from the release from BHK/ABCA1 cells. Experiments were performed in triplicate, and the average values are shown with S.D.

We hypothesized that the two transport substrates of ABCA1, PC and cholesterol, were temporarily sequestered within the ECDs of ABCA1 before being loaded on to apoA-I^17,18^. If this is the case, trypsin treatment of cells, which would cleave ECDs of ABCA1 on the cell surface, may release these sequestered lipids into the medium. To test this hypothesis, we treated BHK/ABCA1 cells, which can be induced to express ABCA1 with the synthetic steroid mifepristone^23^, with increasing concentrations of trypsin for 60 min at 37 °C before measuring the amounts of PC and cholesterol in the medium. The amounts of PC and cholesterol in the medium were elevated following trypsin treatment, reaching a maximum at 50 μg/mL trypsin (Fig. 1). By contrast, much lower amounts of PC and cholesterol were released from mock-transfected cells (BHK/Mock) (Fig. 1). Trypsin treatment for 60 min caused slight increase of LDH release but the expression of ABCA1 did not cause significant cytotoxic effects as verified by an LDH release assay (Supplemental Fig. 1). Furthermore, the trypsin concentration used in this study is less than one-tenth of that normally used for routine cell detachment (0.5–1 mg/mL). These results suggest that PC and cholesterol are temporarily sequestered at trypsin-sensitive sites on the surface of BHK/ABCA1 cells.

### Comparison between trypsin-dependent lipid release and apoA-I–dependent lipid efflux

**Figure 2:**
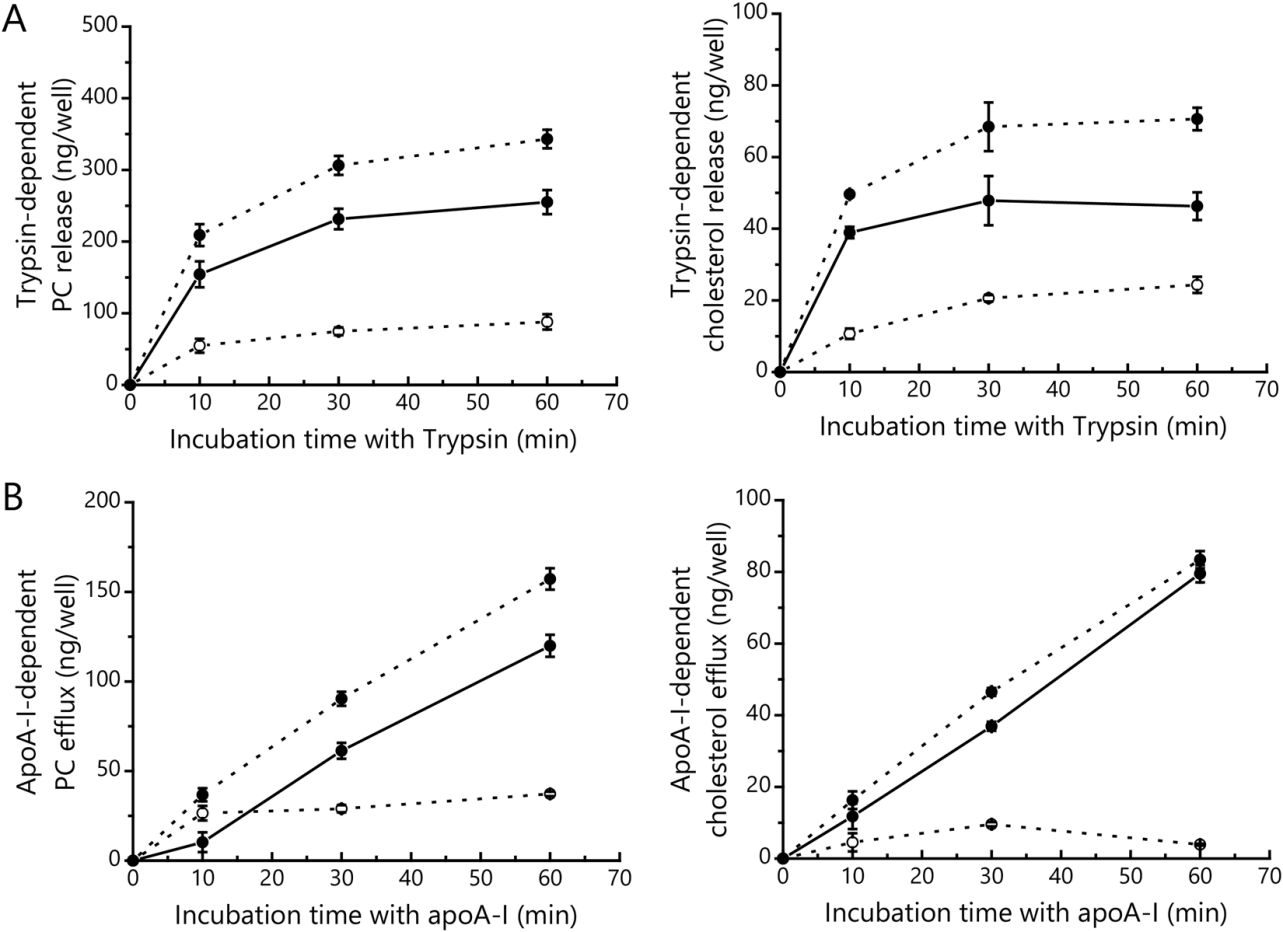
Incubation-time dependence of trypsin-dependent PC and cholesterol release (**A**) and apoA-I–dependent PC and cholesterol efflux (**B**). BHK/Mock (○, dotted line) and BHK/ABCA1 (●, dotted line) cells were incubated with either 50 μg/mL trypsin (A) or 10 μg/mL apoA-I (**B**) for 0, 10, 30, or 60 min at 37 °C. After the incubation, amounts of PC and cholesterol in the medium were measured. ABCA1-specific release of PC and cholesterol (●, solid line) were calculated by subtracting the measured level of release from BHK/Mock cells from the measured level of release from BHK/ABCA1 cells. Experiments were performed in triplicate, and the average values are shown with S.D.

Next, we characterized the time dependence of trypsin-dependent PC and cholesterol release, and compared them with apoA-I–dependent efflux. When BHK/ABCA1 cells were treated with 50 μg/mL trypsin, most of the PC and cholesterol were released within 10 min, and the maximum level of release was observed at 30 min (Fig. 2A). This suggests that the sites in which PC and cholesterol are temporarily sequestered on the cell surface are highly sensitive to trypsin. The specific amounts of PC and cholesterol released at 30 min were 230 ng/well and 47 ng/well, respectively. On the other hand, when BHK/ABCA1 cells were treated with 10 μg/mL apoA-I, the amounts of PC and cholesterol in the medium increased in a linear fashion up until 60 min (Fig. 2B). The apoA-I–dependent PC and cholesterol effluxes measured were 60 ng/well and 37 ng/well at 30 min and 120 ng/well and 80 ng/well at 60 min, respectively. These results suggest that the amount of lipids sequestered in trypsin-sensitive sites on the cell surface are comparable to those loaded on apoA-I within 60 min, while we observed some difference between their cholesterol/PC ratios.

### Trypsin cleaves the ECDs of ABCA1 on the cell surface

**Figure 3:**
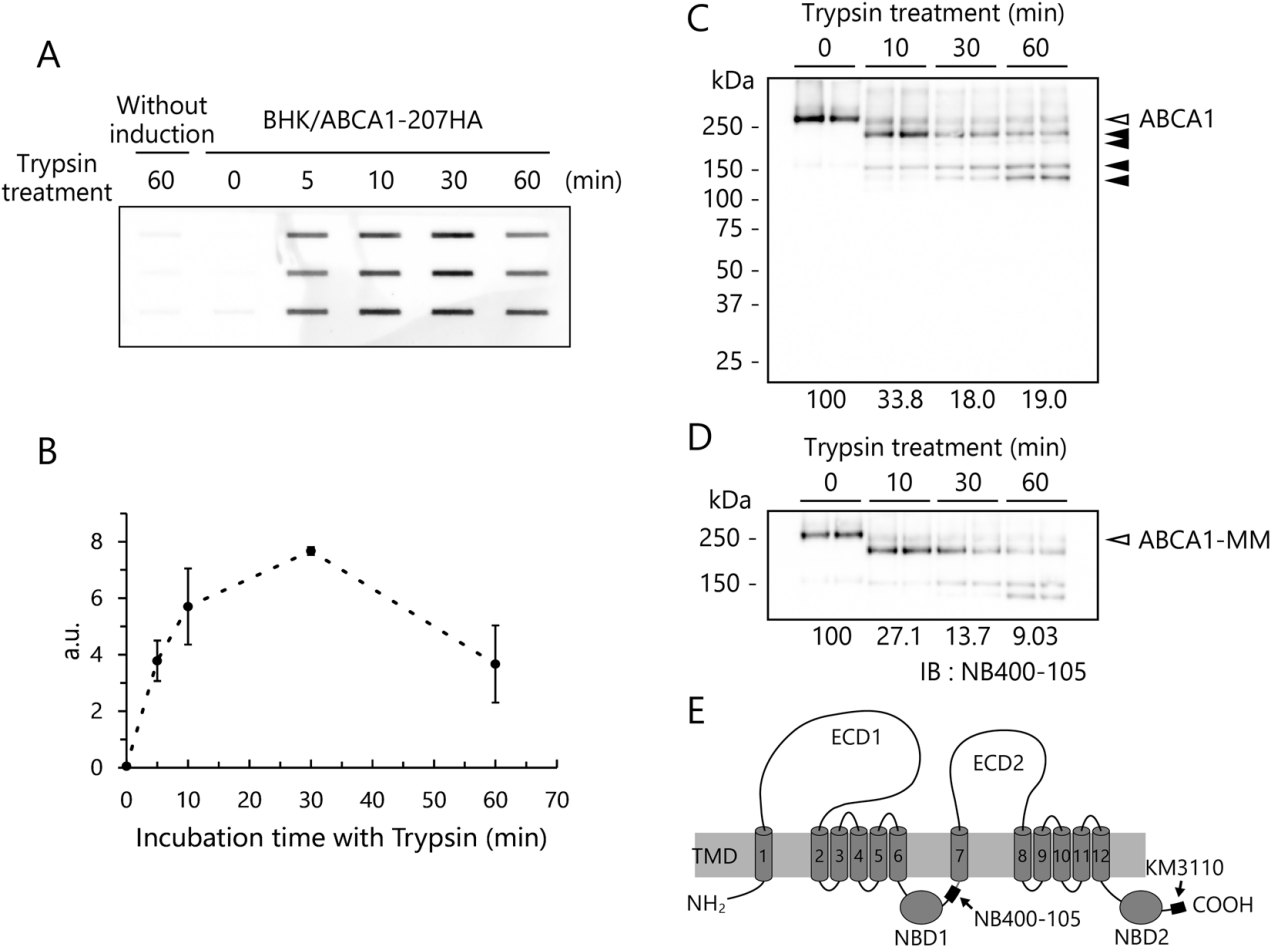
Digestion of ABCA1 on the cell surface by trypsin and release of ECD fragments into the medium. (**A**) BHK/ABCA1-207HA cells after induction with mifepristone were treated with 50 μg/mL of trypsin for 0, 10, 30, or 60 min at 37 °C. Triplicate experiments are shown. The cells without mifepristone induction were also treated with trypsin for 60 min. After trypsin was inactivated, the medium was collected and slot blot analyses of HA-positive peptides in the medium were performed. Similar experiments were performed three times and the representative result was shown. (**B**) Relative intensities of HA-positive signals were calculated. The average values (arbitrary units) are shown with S.D. (**C**) BHK/ABCA1 cells were treated with 50 μg/mL of trypsin for 0, 10, 30, or 60 min at 37 °C. After trypsin was inactivated, surface proteins were biotinylated at 4 °C. Biotinylated proteins (8 μg) were precipitated with monomeric avidin agarose resin and analyzed by western blotting with NB400-105. Full-length ABCA1 and produced fragments are indicated by white and black arrowheads. The average values (relative to the value at 0 min) are shown at the bottom. (**D**) BHK/ABCA1-MM cells were treated with 50 μg/mL of trypsin for 0, 10, 30, or 60 min at 37 °C. After trypsin was inactivated, surface proteins were biotinylated at 4 °C. Biotinylated proteins (8 μg) were precipitated with monomeric avidin agarose resin and analyzed by western blotting with NB400-105. Full-length ABCA1 is indicated by a white arrowhead. The average values (relative to the value at 0 min) are shown at the bottom. (**E**) Schematic diagram of the full-length ABCA1. Recognition sites of two antibodies, NB400-105 and KM3110, are shown (not to scale). The original images of Fig. 3C,D are in Supplemental Fig. 4.

Because trypsin was added to the medium, extracellular domains of membrane proteins, including ECDs of ABCA1, were digested and their fragments would be released to the medium. To examine whether ECDs of ABCA1 were released to the medium by trypsin, BHK cells expressing ABCA1-207HA, in which HA epitope was inserted in ECD1 at position 207, were established. This insertion was reported to have no effects on subcellular localization or functions of ABCA1^17,24,25^. Then, the slot blot analyses were performed to detect fragments of ECD1 by using anti-HA antibody (Fig. 3A,B). The HA-positive signal in the medium rapidly increased until 10 min and the maximal intensity was observed at 30 min. Then the HA-positive signal decreased within 60 min, suggesting the ECD fragments were broken down into small pieces by trypsin after they are released to the medium. These results suggested that most of the surface ABCA1 was cleaved by 50 μg/mL of trypsin in 30 min and that ECD fragments containing HA epitope were released into the medium.

Next, ABCA1 remained on the cell surface was analyzed. BHK/ABCA1 cells were treated with 50 μg/mL of trypsin for the indicated times, and after trypsin was inactivated the remaining membrane proteins on the cell surface were biotinylated. Biotinylated proteins were subsequently precipitated from cell lysate with avidin beads, and ABCA1 was detected by western blotting. The amount of full-length ABCA1 on the cell surface was reduced by 66% and 82% at 10 and 30 min, respectively, and protein fragments of around 220, 200, 150, and 130 kDa were detected using the polyclonal antibody NB400-105, which was raised against the linker region that joins the two halves of ABCA1 (Fig. 3C). ABCA1 fragments of around 220, 200, and 70 kDa were also detected by western blotting of whole-cell lysates with the monoclonal antibody KM3110, which was raised against the C-terminal 20 amino acids of ABCA1 (Supplemental Fig. 2). These results suggest that the ECDs of ABCA1 on the cell surface are highly sensitive to trypsin and are cleaved at some specific sites in a time-dependent manner. The time course of the disappearance of full-length ABCA1 from the cell surface coincides with those of the appearance of the HA-positive signal in the medium (Fig. 3A,B) and of trypsin-dependent lipid release into the medium described in the previous section (Fig. 2).

### PC and cholesterol release into the medium by trypsin is dependent on the ATPase activity of ABCA1

**Figure 4:**
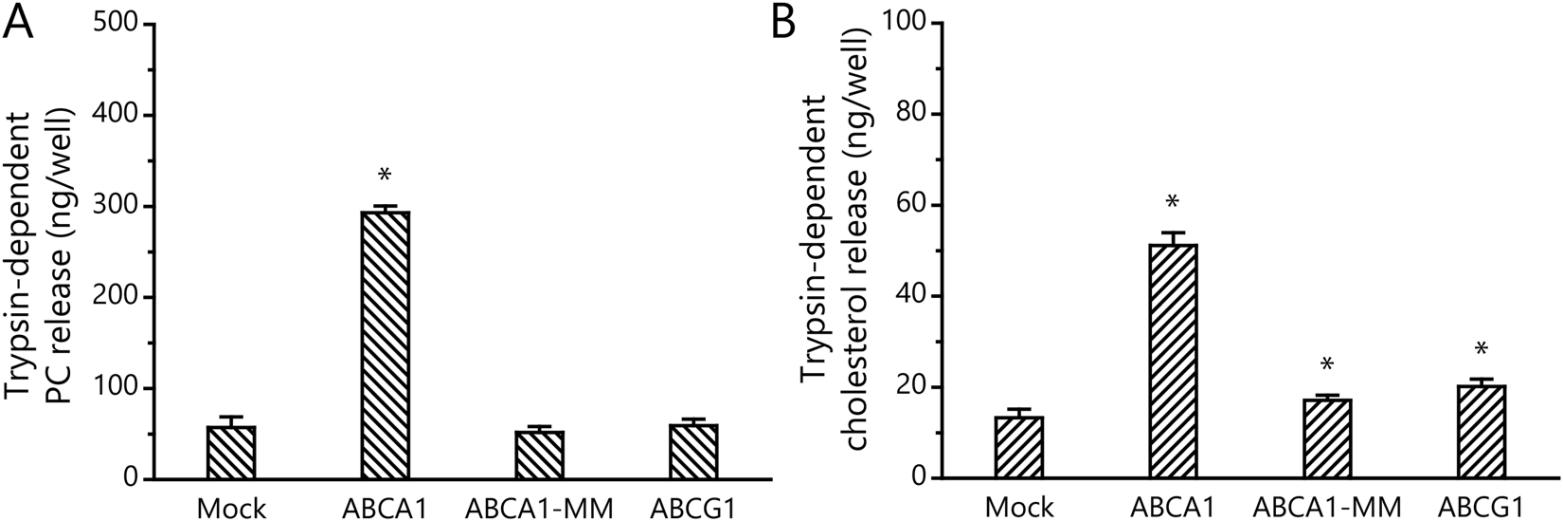
Trypsin-dependent PC and cholesterol release from BHK/ABCA1-MM and BHK/ABCG1. BHK/Mock, BHK/ABCA1, BHK/ABCA1-MM, and BHK/ABCG1 cells were treated with either 0 or 50 μg/mL of trypsin for 60 min at 37 °C. Amounts of PC and cholesterol released by the trypsin treatment were calculated. Experiments were performed in triplicate, and average values are shown with S.D. *P < 0.01 compared with mock.

ApoA-I–dependent lipid efflux and the structural changes of ECDs, which could be caused by lipid accumulation within their ECDs, are both dependent on ABCA1 ATPase activity. Hence, to verify that PC and cholesterol release induced by trypsin treatment is dependent on ABCA1 function, we established BHK cells that express ABCA1-MM (BHK/ABCA1-MM), in which two lysine residues critical for ATP hydrolysis are replaced by methionines^26^. The ECDs of ABCA1-MM were cleaved by trypsin similarly to those of wild-type ABCA1 (Fig. 3D). However, trypsin treatment of BHK/ABCA1-MM released neither cholesterol nor PC into the medium (Fig. 4), as in the case of apoA-I treatment (Supplemental Fig. 3). These results suggest that PC and cholesterol are sequestered in trypsin-sensitive sites on the cell surface by ABCA1 in an ATP hydrolysis–dependent manner.

### Trypsin treatment of BHK/ABCG1 cells does not cause lipid release into the culture medium

We next examined whether trypsin treatment of cells expressing ABCG1, which exports cholesterol and choline-phospholipids like ABCA1^27^, but does not have large ECDs, would cause lipid release into the medium. When BHK/ABCG1 cells were treated with 50 μg/mL trypsin for 60 min at 37 °C, no cholesterol was released into the medium (Fig. 4), whereas HDL-dependent cholesterol efflux was observed, as reported previously^27^ (Supplemental Fig. 3). These results suggest that trypsin-sensitive lipid-sequestered sites on the cell surface are not generated by ABCG1.

### Estimation of amount of PC and cholesterol sequestered by each ABCA1 molecule

**Figure 5:**
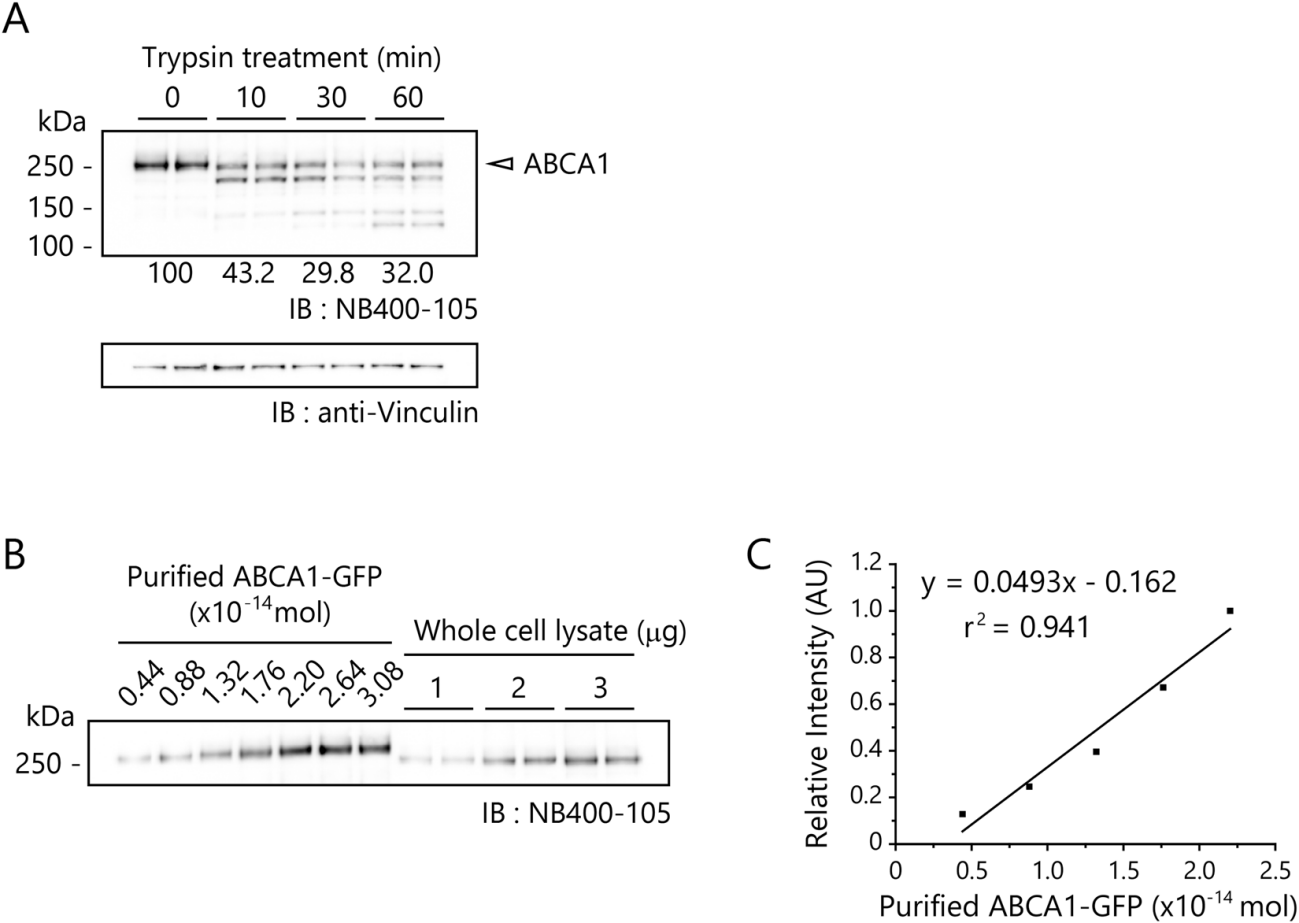
Quantification of ABCA1 on plasma membrane of BHK/ABCA1 cells. (**A**) BHK/ABCA1 cells were treated with 50 μg/mL of trypsin for 0, 10, 30, or 60 min at 37 °C. After trypsin was inactivated, cells were lysed, total cellular proteins (4 μg) were resolved on SDS-polyacrylamide gels, and ABCA1 was detected by western blotting with NB400-105. Full-length ABCA1 is indicated by a white arrowhead. The average values of full-length ABCA1 (relative to the value at 0 min) are shown at the bottom. Vinculin is shown as a loading control. (**B**) Quantitative comparison of purified ABCA1-GFP and ABCA1 in whole-cell lysates. Proteins were resolved on 5–20% gradient SDS-polyacrylamide gels, and ABCA1 was detected by western blotting with NB400-105. Experiments were performed in duplicate, and representative data are shown. (**C**) Standard curve obtained from Fig. 4B. Because band intensity seemed to be saturated at 2.20 × 10^−14^ mol of ABCA1, the standard curve was generated from data obtained with lower amounts of protein. Relative intensities, normalized to the intensity at 2.20 × 10^−14^ mol of ABCA1, are shown. Experiments were performed in duplicate, and representative data are shown. The original images of Fig. 5A,B are in Supplemental Fig. 4.

Finally, we estimated the amount of lipid sequestered by each ABCA1 molecule at trypsin-sensitive sites on the cell surface. First, we quantitated the amount of ABCA1 in whole-cell lysate by comparing it with purified ABCA1 (Fig. 5C,D). Our western-blot analysis suggested that 0.54 × 10^−14^ mol of ABCA1 are present in 1 μg of whole cell protein. Because 140 μg of protein was obtained from each well, the amount of ABCA1 in whole-cell lysate was 0.76 pmol/well. Trypsin treatment reduced the amount of full-length ABCA1 in whole-cell lysate by 57%, 70%, and 68% after 10, 30, and 60 min, respectively (Fig. 5A,B). Therefore, the amount of ABCA1 digested during the trypsin treatment for 30 min was 0.76 × 0.70 = 0.53 pmol/well (3.2 × 10^11^ molecules/well). Because the amounts of PC and cholesterol released from BHK/ABCA1 were 230 ng and 47 ng/well (Fig. 2A), respectively, we calculated that 560 molecules of PC and 230 molecules of cholesterol are sequestered at trypsin-sensitive sites on the cell surface by each ABCA1 molecule.

## Discussion

In this study, we found that trypsin treatment caused the rapid release of PC and cholesterol from BHK/ABCA1 cells, which suggests that ABCA1 temporarily sequesters PC and cholesterol in trypsin-sensitive sites on the cell surface before being loaded onto apoA-I. Several lines of evidence obtained in this study support the hypothesis that the trypsin-sensitive sites on the cell surface are the large ECDs of ABCA1, and that ABCA1 temporarily sequesters transported lipids within its ECDs during nascent HDL formation.

Because the trypsin concentration (50 μg/mL) used in this study was much lower than that normally used for detaching cells (0.5–1 mg/mL), and because it caused no cell detachment after 30 min, we believe that surface proteins involved in cell attachment were not affected by trypsin at 50 μg/mL. However, the ECDs of ABCA1, consisting of more than 900 amino-acid residues, were cleaved rapidly by trypsin (50 μg/mL) at some specific sites, which suggests that the ECDs of ABCA1 are highly sensitive to trypsin. Intriguingly, the time courses of trypsin-dependent release of ECD fragments and of trypsin digestion of ABCA1 on the cell surface (66% and 82% were cleaved at 10 min and 30 min, respectively) coincided with that of lipid release (Figs 2 and 3).

**Figure 6:**
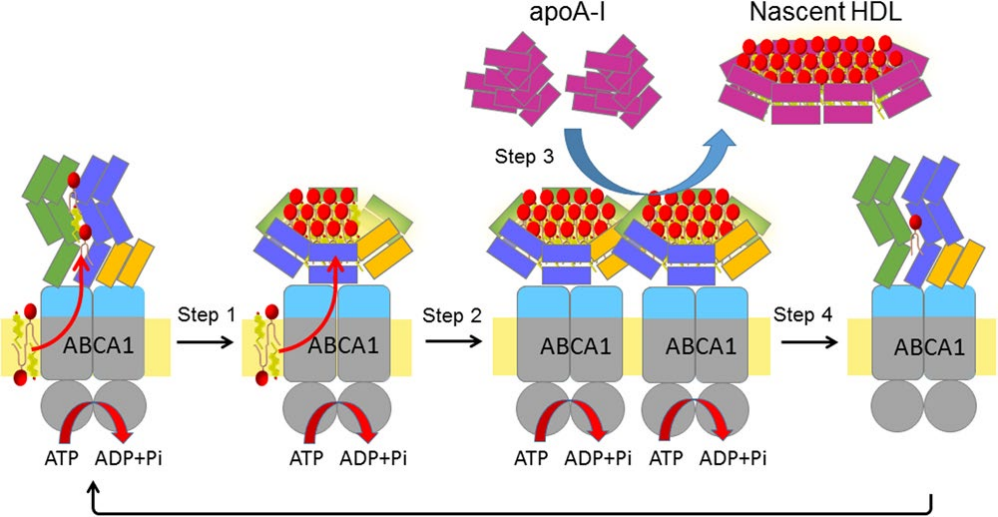
Schematic illustration of nascent HDL generation by ABCA1. ABCA1 monomers translocate PC and cholesterol in an ATP-dependent manner and sequester them within ECDs (Step 1). When enough PC and cholesterol have been sequestered by the ECDs, the ECDs undergo conformational changes that lead to dimer formation and to the halting of their diffusive movement (Step 2). Lipid-free apoA-I molecules may bind directly to the ECDs of ABCA1 dimers (Step 3), and become loaded with lipids sequestered within the ECDs. Nascent HDL is generated and the ABCA1 dimer dissociates into monomers upon releasing the sequestered lipids (Step 4), thereby returning to Step 1.

The trypsin-dependent lipid release we observed does not occur in cells expressing ABCG1, which exports cholesterol and choline-phospholipids like ABCA1^27^ but does not have large ECDs. Although it is controversial where ABCG1 functions in the cell^28–30^, ABCG1 localizes to the plasma membrane to function when expressed in BHK cells and HEK293 cells^27,31,32^. This suggests that large ECDs are necessary for keeping lipids within trypsin-sensitive lipid sequestration sites on the cell surface. We have previously reported that the ECDs of ABCA1 undergo large conformational changes in an ATP-dependent manner^17^, and that these conformational changes are possibly due to the temporary sequestration of transported lipids. The ECDs of ABCA1 consist of more than 900 amino-acid residues, much larger than the apoA-I molecule, which consists of 243 amino-acid residues. Because the ECDs of ABCA1, like apoA-I, are predicted to contain several amphipathic α-helices according to the software JPred 4^33^, a bilayer-like complex of PC and cholesterol may be sequestered by wrapping them with the ECDs’ amphipathic α-helices as illustrated in Fig. 6.

We tried to isolate fragments of ECDs associated with the released lipids by using OptiPrep gradient centrifugation, glycerol gradient centrifugation and a gel filtration. However, they were detected only in large aggregates (data not shown). This suggests that the ECDs of ABCA1 are cleaved at some specific sites by trypsin when they are correctly folded on the plasma membrane and limited digested fragments are released to the medium (as shown Fig. 3, until 30 min). However, once they were released to the medium, ECD fragments were broken down into small pieces by trypsin, because ABCA1 ECDs are rich in basic amino acid residues and contain more than 90 trypsin-cleavable sites. Probably they formed aggregates with fragments released from other membrane proteins and also with released lipids.

The time course of trypsin-dependent PC release and cholesterol release were quite similar to each other (Fig. 2A), suggesting that PC and cholesterol are sequestered at the same trypsin-sensitive sites on the cell surface. We estimated that the number of ABCA1 molecules digested by trypsin on the cell surface was 3.2 × 10^11^/well (6.4 × 10^5^/cell). The amounts of PC and cholesterol released from BHK/ABCA1 were 230 and 47 ng/well, respectively (Fig. 2A). We therefore calculated that 560 molecules of PC and 230 molecules of cholesterol were sequestered by each ABCA1 molecule. Duong *et al*. reported that a 9-nm discoidal HDL particle contains 192 molecules of PC and 24 molecules of cholesterol, and a 12-nm discoidal HDL particle contains 585 molecules of PC and 117 molecules of cholesterol^3^. Therefore, the estimated amounts of PC and cholesterol sequestered by each ABCA1 molecule are almost enough to generate a 12-nm discoidal HDL particle.

The experiments of trypsin-dependent lipid release and apoA-I–dependent lipid efflux whose results are shown in Fig. 2 were performed after mifepristone was removed as described in Materials and Methods. However, new ABCA1 protein would be produced and appear at the surface after mifepristone was removed, because some ABCA1 mRNA persists in cells for a while. Furthermore, ABCA1 generates HDL repeatedly in an apoA-I dependent manner. In contrast, trypsin digests all the surface ABCA1 immediately and thereby halts ABCA1 function. Therefore, it is difficult to compare the amount of lipid sequestered at trypsin-sensitive sites with that loaded onto apoA-I. However, it is possible to compare their cholesterol/PC ratios, and we observed differences in these ratios. Therefore, it is likely that apoA-I does not acquire all the lipids sequestered at trypsin-sensitive sites. However, because single-molecule imaging using TIRF microscopy revealed that ABCA1 forms a dimer before interacting with apoA-I^18^, the ECDs of ABCA1 dimers could sequester a sufficient amount of lipids to generate nascent HDL. Because ABCA1 generates two types (9 nm and 12 nm in diameter) of discoidal HDL whose cholesterol/PC ratios are quite different^3^ as described above and because the ratio of generated 9-nm and 12-nm discoidal HDL would vary, it may be difficult to compare the cholesterol/PC ratios of trypsin-dependent lipid release and apoA-I–dependent lipid efflux.

The observations we made in our study could not conclusively support the direct loading model or exclude the indirect model. However, because plasma membrane proteins (including ABCA1) are thought to be excluded from the exovesiculated membrane domains created by ABCA1 in the indirect model^34^, that model cannot explain our observations. Only one candidate has been reported to connect secreted lipids with the cell, namely the extracellular matrix. Jin, X. *et al*.^35,36^ reported that significant extracellular depots of lipid secreted from macrophages are associated with the extracellular matrix, and that lipids are released by trypsin treatment. However, they also showed that ABCG1, like ABCA1, mediates cholesterol deposition in the extracellular matrix. Because we have shown in this study that trypsin-dependent lipid release does not occur in cells that express ABCG1, the mechanism proposed by Jin *et al*. would not explain our observations. Furthermore, the trypsin concentration used by Jin *et al*. to digest the extracellular matrix was 2.5% (2.5 mg/ml), 50 times more than what we used in this study (50 μg/ml). The cholesterol deposition observed in the extracellular matrix could be specific to macrophages because we could barely detect cholesterol deposition in the extracellular matrix in the case of BHK cells expressing ABCA1 (data not shown).

Qian, H. *et al*. recently reported the cryo-EM structure of human ABCA1 at 4.1 Å resolution^37^. The overall structure resembles an elongated torch about 200 Å in height and the ECDs form the upward-flaring flame that is about 100 Å high. The two helical domains and the α-helical hairpin structure in each ECD constitute a hydrophobic tunnel, which may serve either as temporary storage or as a delivery passage for lipids. Twelve out of 17 α-helices forming the hydrophobic tunnel show a strong amphipathic nature, like the ten α-helices of apoA-I. The reported structure supports our hypothesis that PC and cholesterol are temporarily sequestered by wrapping them with the amphipathic α-helices of the ECDs (Fig. 6). We have reported the accessibility of an antibody to the peptide inserted at position 443 is reduced in an ATP-dependent manner^17^. Interestingly, residue 443 is located at the distal end of the helical domain II, suggesting that large conformational changes occur in helical domains in an ATP-dependent manner. Although apparently several hundreds of lipid molecules cannot be stored in the hydrophobic tunnel of the reported structure of ABCA1, the large conformational changes could make it possible.

ApoA-I directly interacts with the ECDs of ABCA1^12,14,15^. Therefore, lipid sequestered by the ECDs could be loaded on to apoA-I while it is bound to the ECDs. Based on our imaging analysis^18^, we propose the following model for nascent HDL generation (Fig. 6). ABCA1 monomers that have not sequestered sufficient lipids constantly translocate lipids instead, acting as ATP-dependent transporters, and diffuse freely in the plasma membrane (step 1). ABCA1 sequesters cholesterol and PC within its large ECDs, and undergoes conformational changes^17^ leading to dimer formation. The lipidated ABCA1 dimers interact with membrane–skeletal actin filaments and other stable structures in the plasma membrane, leading to the halting of their diffusive movement, and become ready for apoA-I access (step 2). Lipid-free apoA-I directly binds to the ECD(s) of the ABCA1 dimers (step 3), and becomes loaded with lipids sequestered by ABCA1 (step 4). Upon releasing the sequestered lipids, the ABCA1 dimer dissociates into monomers and is released from immobilization, and also resumes diffusion in the plasma membrane, again sequestering lipids by constantly translocating them in an ATP-dependent manner (step 1). When ABCA1 forms dimers, huge structures comprising ECDs that bind more than 1000 lipid molecules each are likely to form on the cell surface. Such a huge structure may cause the diffusive movement of ABCA1 to halt.

In summary, our results suggest that ABCA1 dimers on the cell surface sequester cholesterol and PC within their ECDs, and that apoA-I molecules, which can interact with the ECDs, may become loaded with sequestered lipids to form discoidal HDL. We believe that this process constitutes the main pathway for nascent HDL formation.

## Materials and Methods

### Materials

Anti-ABCA1 monoclonal antibody KM3110 was generated against the C-terminal 20 amino acids of ABCA1 in mice^38^. Rabbit polyclonal anti-ABCA1 antibody (NB400-105) was obtained from Novus Biologicals. Mouse monoclonal anti-HA antibody (sc7392) was obtained from Santa Cruz Biotechnology. Mouse monoclonal anti-vinculin antibody (V9131), trypsin from bovine pancreas, choline oxidase, FLAG-M2 agarose resin, 1 × FLAG peptide, and 3 × FLAG peptide were purchased from Sigma-Aldrich. Soybean trypsin inhibitor was purchased from Nacalai Tesque. Phospholipase D was purchased from Enzo Life Sciences. Cholesterol oxidase and horseradish peroxidase (HRP) were purchased from Oriental Yeast. Amplex Red was purchased from Molecular Probes. As for PC and cholesterol standards, we used the PC and cholesterol assay kit from Wako. Ez-link Sulfo-NHS-Biotin and monomeric avidin agarose were purchased from Thermo-Fisher Scientific. Gradient gels (5–20%) (e-PAGEL) were purchased from ATTO. Cholesteryl hemisuccinate (CHS) was purchased from Anatrace. Tris(2-carboxyethyl)phosphine was purchased from Tokyo Chemical Industry. Protease inhibitor cocktail was purchased from Roche Diagnostics. CHAPS was purchased from Dojindo. Ultrafree 0.22 μm filter and Amicon Ultra 100 K were purchased from Merck Millipore. Recombinant apoA-I was prepared as reported previously^39^. HDL was purchased from Calbiochem.

### Cell culture

BHK/Mock, BHK/ABCA1, and BHK/ABCG1 cells^23,32^ were kindly provided by the late Dr. John Oram and Dr. Chongren Tang of the University of Washington. In these cell lines, protein expression can be strongly induced by adding the synthetic steroid, mifepristone (GeneSwitch system, Invitrogen). Cells expressing ABCA1-MM, which has two lysine residues (K939 and K1952) crucial for ATP hydrolysis replaced by methionine^26^, and cells expressing ABCA1-207HA, which has the influenza virus hemagglutinin (HA) epitope sequence (coding YPYDVPDYA) between G207 and D208^40^, were generated as follows. Human ABCA1-MM and ABCA1-207HA cDNA were inserted into pGene/V5-HisA (blasticidin), in which the original zeocin resistance gene was replaced by the blasticidin resistance gene. BHK/pSwitch cells were transfected with pGene/V5-HisA (blasticidin)/ABCA1-MM or ABCA1-207HA, and the stably transfected cell lines were subject to selection with 350 μg/mL hygromycin and 5 μg/mL blasticidin. These cells were cultured in Dulbecco’s modified Eagle’s medium (DMEM) containing 10% fetal bovine serum at 37 °C under 5% CO_2_. FreeStyle 293-F cells were maintained in FreeStyle 293 Expression Medium containing 5 μg/mL gentamicin at 37 °C under 8% CO_2_.

### Treatment with either trypsin or apoA-I

BHK cells were plated on 24-well plates at a density of 1.5 × 10^5^ cells/well. After incubating at 37 °C for 24 h, the culture medium was replaced by DMEM containing 0.02% bovine serum albumin (BSA) and 10 nM mifepristone, and incubated for an additional 24 h. Cells were washed twice with 500 μL Hank’s balanced salt solution (HBSS), and HBSS containing trypsin or apoA-I was added to the cells before they were incubated for the indicated time at 37 °C. In the case of trypsin, trypsin inhibitor dissolved in HBSS was added to the medium at a final concentration of 190 μg/mL. Experiments were done at least twice.

### PC and cholesterol measurement

PC and cholesterol measurements were based on previous reports^41,42^. Culture medium was transferred to a black 96-well plate, which was incubated with 1 mM CaCl_2_ and 10 U/mL phospholipase D for 30 min at 37 °C for PC measurement. Medium was subsequently incubated for 30 min at 37 °C with 1 U/mL choline oxidase (for PC measurement), 2 U/mL cholesterol oxidase (for free cholesterol measurement), 2 U/mL HRP, and 100 μM Amplex Red in phosphate-buffered saline (PBS) containing 0.01% Triton X-100 and 5 mM cholate. After incubation, fluorescence intensity (Ex/Em = 535/590) was measured using a microplate reader (Infinite F200, TECAN).

### LDH release assay

Cells were plated on a 24-well plate at 1.0 × 10^5^ cells/well, and incubated for 24 h at 37 °C. Cell were washed twice with DMEM before being treated with either 0.1% DMSO or 10 nM mifepristone for 16 h at 37 °C. Cells were washed twice with HBSS, and treated with 50 μg/mL trypsin for the indicated time at 37 °C. Trypsin inhibitor (same amount as trypsin) dissolved in HBSS was added to the medium. Medium was transferred to 96-well plates. Cells were lysed with 1% Triton X-100/PBS, and the lysates were transferred to 96-well plates. The amount of LDH was measured using a cytotoxicity assay kit (Cytotox96, Promega).

### Slot blotting

BHK/ABCA1-207HA cells were treated with 50 μg/mL trypsin for the indicated time at 37 °C, and the medium was collected. Samples were transferred to PVDF membrane by Bio-Dot SF(BIO-RAD) and analyzed with anti-HA antibody.

### Western blotting

Cells were lysed with lysis buffer (20 mM Tris-HCl buffer, pH 7.5) containing 1% Triton X-100, 0.1% sodium dodecyl sulfate (SDS), 1% sodium deoxycholate, and protease inhibitors (100 μg/ml p-amidinophenyl)methanesulfonyl fluoride, 10 μg/ml leupeptin, and 2 μg/ml aprotinin). Samples were electrophoresed on 5–20% gradient SDS-polyacrylamide gels and analyzed by western blotting with the indicated antibodies.

### Biotinylation and isolation of ABCA1 from plasma membrane

Cells were washed twice with ice-cold PBS containing 0.1 mg/ml CaCl_2_ and 0.1 mg/ml MgCl_2_∙6H_2_O and reacted with 0.5 mg/mL of Sulfo-NHS-Biotin at 4 °C for 30 min in the dark. The cells were subsequently washed five times with ice-cold Tris-buffered saline containing 0.1 mg/ml CaCl_2_ and 0.1 mg/ml MgCl_2_∙6H_2_O, and lysed with lysis buffer. Monomeric avidin agarose resin equilibrated with lysis buffer was added to the lysate at a ratio of 20 μL resin to 40 μg protein, and samples were rotated for 3 h at 4 °C. Resin was washed three times with lysis buffer containing 150 mM NaCl.

### Purification of human ABCA1

FreeStyle 293-F cells were transfected with 1 μg/mL of pcDNA3.1(−)/ABCA1-GFP-FLAG plasmid using 2.5 μg/mL polyethyleneimine “MAX” (Polyscience)^43^. After 48 h incubation, cells were collected and solubilized with buffer A (50 mM HEPES (pH 7.4), 150 mM NaCl, 1 mM MgCl_2_, 10% glycerol, 1 mM EDTA, 1 mM Tris(2-carboxyethyl)phosphine, and 0.002% CHS) containing 18 mM CHAPS and protease inhibitor cocktail for 45 min at 4 °C in a constantly rotated tube. The cell lysate was centrifuged (100,000 *g*, 10 min, 4 °C) to pellet cells that were not solubilized. Solubilized proteins were applied to FLAG-M2 agarose resin pre-equilibrated with buffer A containing 10 mM CHAPS, and the mixture was rotated constantly for 18 h. The resin was washed six times with five bed volumes of buffer A containing 10 mM CHAPS. The protein was eluted from the resin with 2 bed volumes of buffer A containing 10 mM CHAPS, 300 μg/mL 1 × FLAG peptide, and 3 × FLAG peptide. The eluate was obtained by removing the resin using Ultrafree 0.22 μm filters, and subsequently concentrated by centrifugation using an Amicon Ultra 100 K to the desired protein concentration. All purification steps were performed at 0–4 °C. The amount of purified ABCA1-GFP was estimated by comparison with BSA on SDS-polyacrylamide gel stained with Coomassie Brilliant Blue R-250.

### Statistical analysis

The statistical significance of differences between mean values was analyzed using the unpaired t-test. Multiple comparisons were performed using the Tukey test following ANOVAOneWay.

## Electronic supplementary material

Supplementary information
