# Supplementary material for: Temporary sequestration of cholesterol and phosphatidylcholine within extracellular domains of ABCA1 during nascent HDL generation
Source: Sci Rep. 2018 Apr 18;8:6170. doi: 10.1038/s41598-018-24428-6 (PMC5906560; doi:10.1038/s41598-018-24428-6)
Supplement: Supplementary file 1 — Supplementary information [file 41598_2018_24428_MOESM1_ESM.pdf]

Supplementary Information for “Temporary sequestration of cholesterol and phosphatidylcholine within extracellular domains of ABCA1 during nascent HDL generation” by Masato Ishigami, Fumihiko Ogasawara, Kohjiro Nagao, Hidehiko Hashimoto, Yasuhisa Kimura, Noriyuki Kioka and Kazumitsu Ueda

Supplemental Figure 1. LDH release from BHK cells expressing ABCA1 following trypsin treatment. Cells were first treated with either DMSO (empty bars) or 10 nM mifepristone (black bars) for 16 h at 37°C, and subsequently with 50 μg/mL trypsin for the indicated time at 37°C. The amounts of LDH released into the medium and remaining in the cell lysate were measured using a cytotoxicity assay kit (Cytotox96, Promega). Released LDH at the indicated time points, as a percentage of total LDH, is shown.

Supplemental Figure 2. Analysis of ABCA1 and its trypsin-digested products by western blotting using KM3110. BHK/A1 cells were treated with 50 μg/mL of trypsin for either 0, 10, 30, or 60 min at 37°C. Cells were lysed and analyzed by SDS-polyacrylamide gel electrophoresis using 5–20% gradient gel, and then ABCA1 was detected by western blotting with KM3110. Full-length ABCA1 and peptide fragments of ABCA1 are indicated by white and black arrowheads, respectively. Asterisk indicates a non-specific band. The original images are in Supplemental figure 4.

Supplemental Figure 3. ApoA-I and HDL-dependent cholesterol and PC efflux from BHK/ABCA1-MM and BHK/ABCG1. BHK/Mock, BHK/ABCA1, BHK/ABCA1-MM, and BHK/ABCG1 cells were each incubated with either 10 μg/mL apoA-I or 20 μg/mL human plasma HDL for 60 min at 37°C. The amounts of PC and cholesterol exported into the medium were measured by fluorescence enzyme assay. Experiments were performed in triplicate, and average values are shown with S.D. *P<0.01 compared with mock.

Supplemental Figure 4. Original images for Figs. 3, Fig. 5 and Supplemental fig.2.

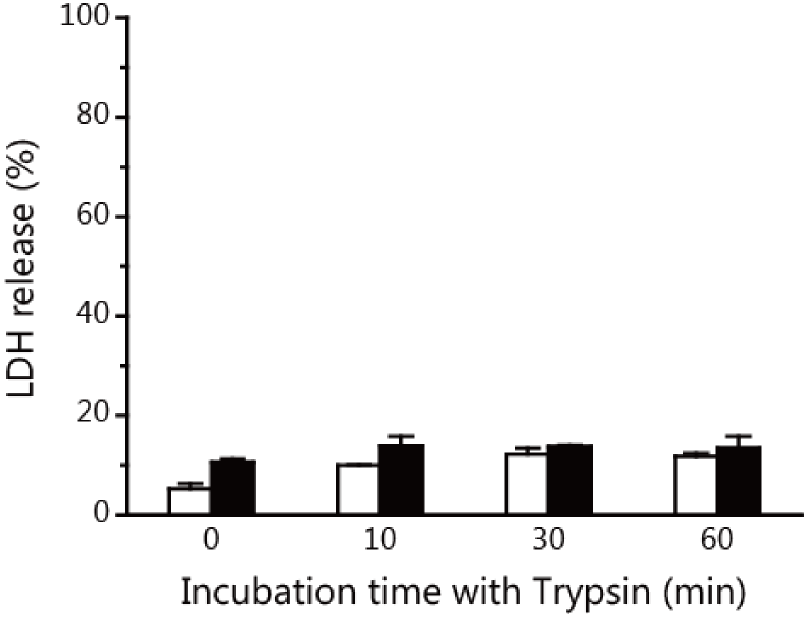

Supplemental Figure 1

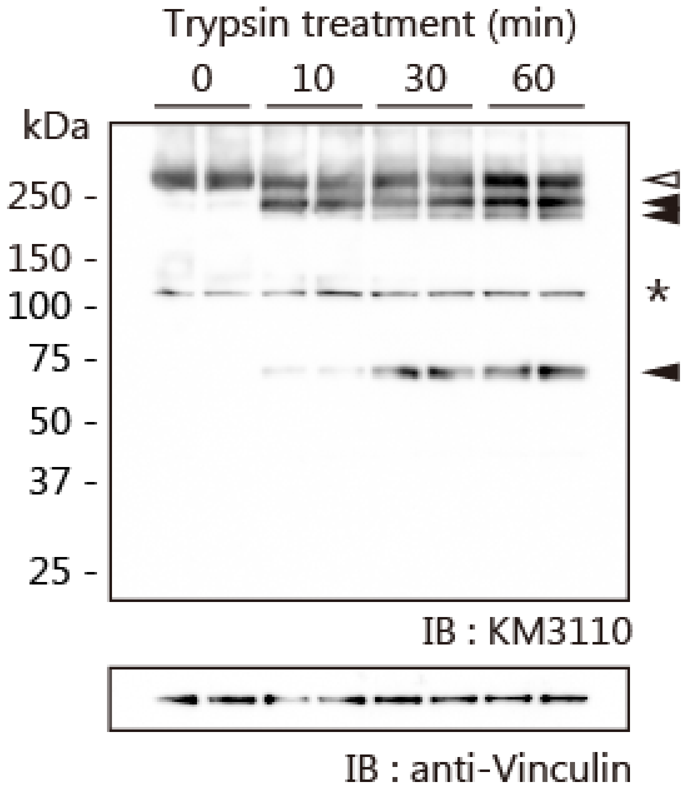

Supplemental Figure 2

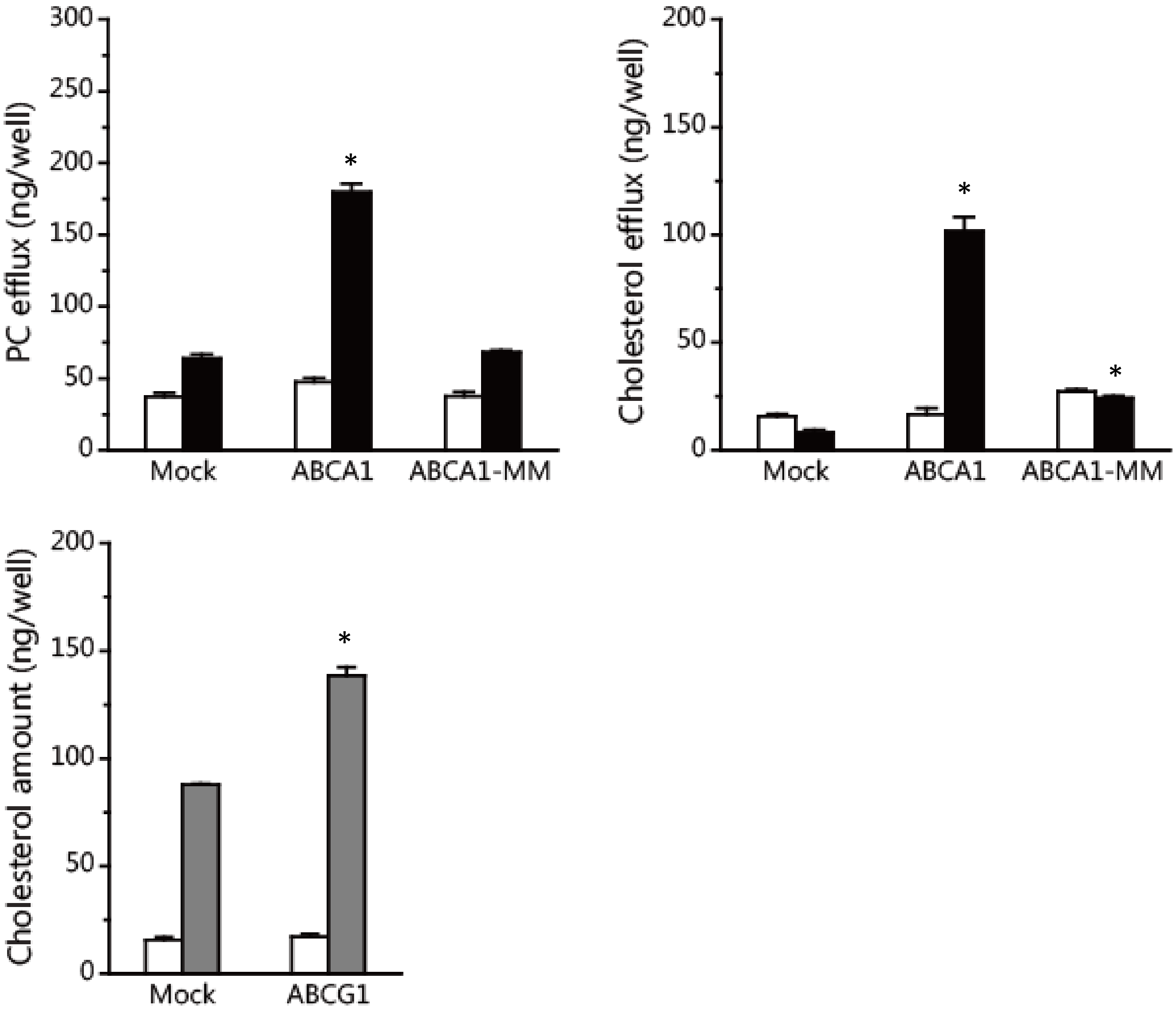

Supplemental Figure 3

Fig. 3C

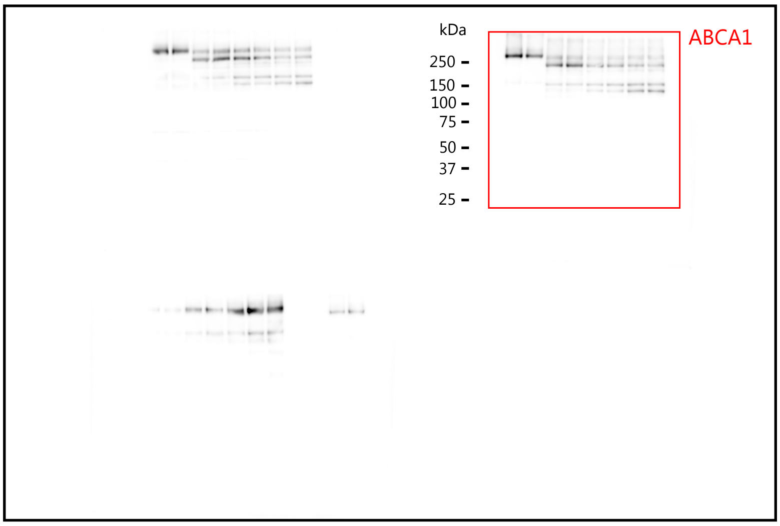

Fig. 3D

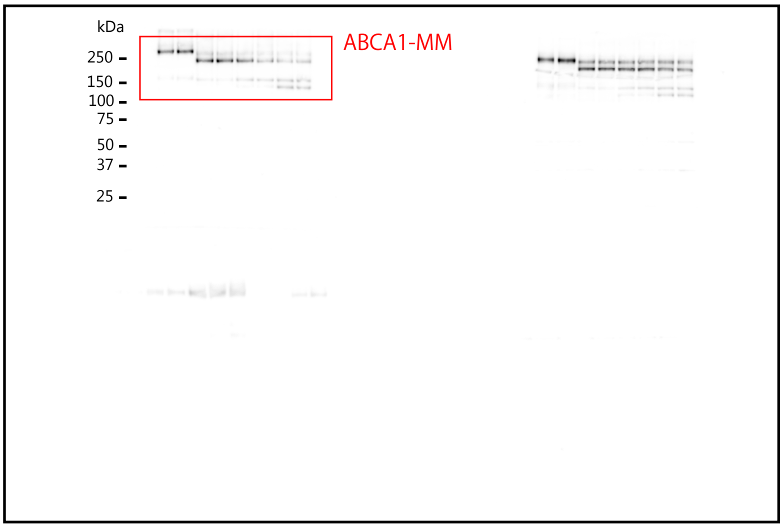

Fig. 5A/C

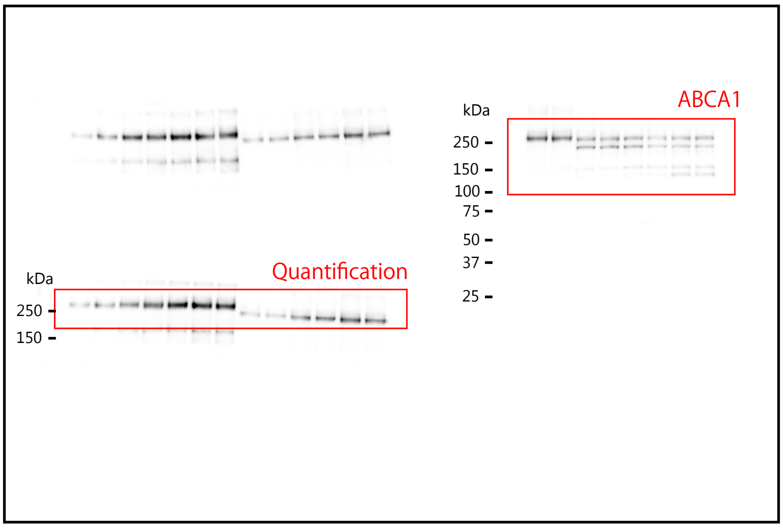

Fig. 5A

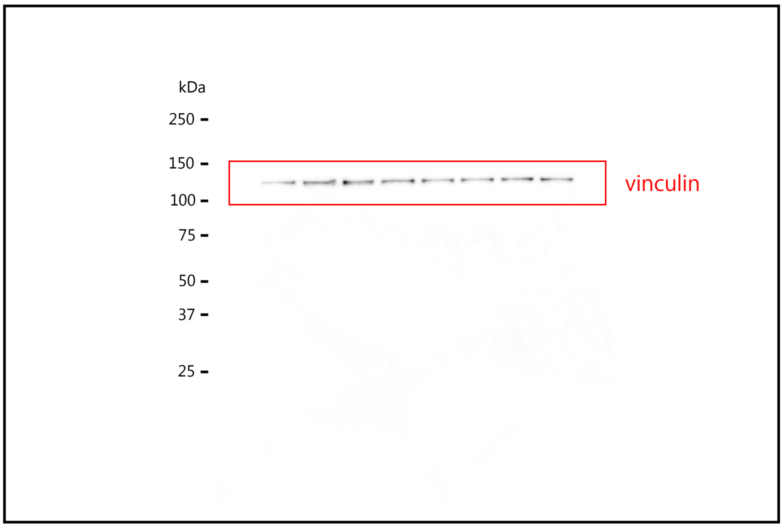

Suppl fig. 2

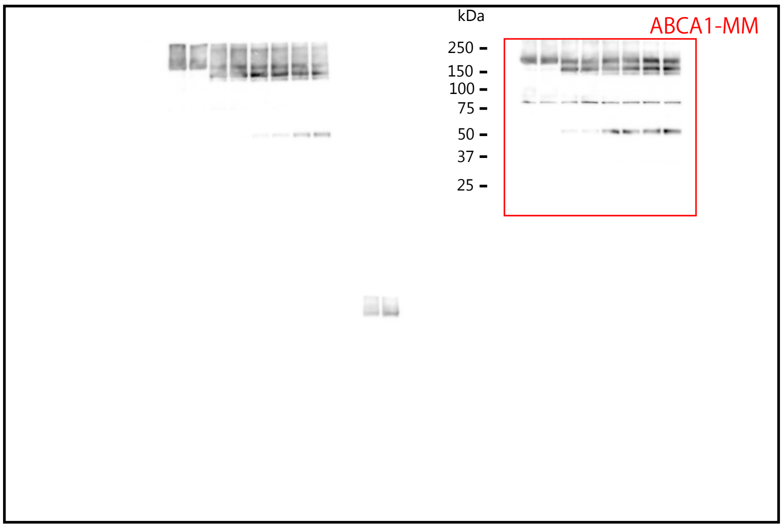

Suppl fig. S2

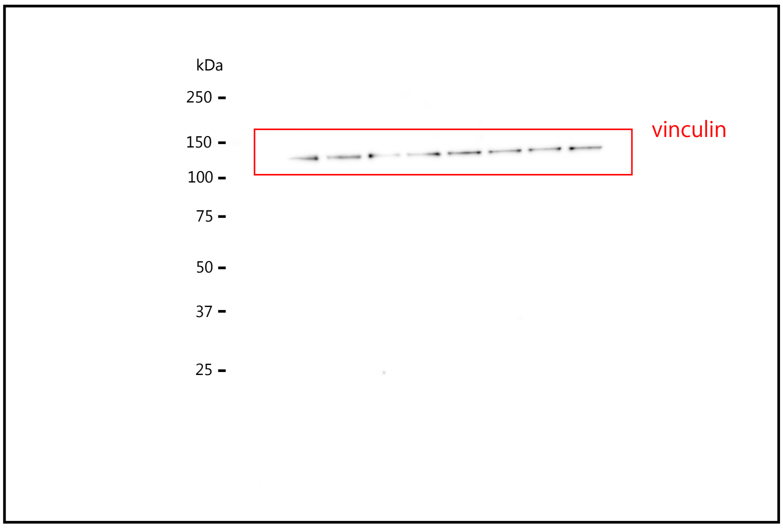

Supplmental Figure 4
